# The proteasome inhibitor bortezomib attenuates renal fibrosis in mice via the suppression of TGF-β1

**DOI:** 10.1038/s41598-017-13486-x

**Published:** 2017-10-12

**Authors:** Moko Zeniya, Takayasu Mori, Naofumi Yui, Naohiro Nomura, Shintaro Mandai, Kiyoshi Isobe, Motoko Chiga, Eisei Sohara, Tatemitsu Rai, Shinichi Uchida

**Affiliations:** Department of Nephrology, Graduate School of Medical and Dental Sciences, Tokyo Medical and Dental University, Tokyo, Japan

## Abstract

Kidney fibrosis and fibrogenesis significantly exacerbate chronic kidney disease (CKD) progression and are essential therapeutic targets. Bortezomib (BZM) is a proteasome inhibitor used for the treatment of multiple myeloma (MM). Several studies have demonstrated that BZM attenuates renal impairment in patients with MM, although this effect is generally considered to be the result of MM remission. Recently, several studies on BZM reported anti-fibrotic effects on liver and skin in experimental animal models. However, its effect on renal fibrosis has yet to be examined. Here, we investigated the anti-fibrotic effects of BZM in an experimental mouse model of fibrosis that uses aristolochic acid I (AA). Ten weeks of AA administration with BZM treatment twice a week significantly attenuated AA-induced renal dysfunction and albuminuria, reduced the expression of renal fibrosis-related proteins and kidney injury markers, such as αSMA, Kim1, and Ngal, and prevented renal fibrosis at the level of histopathology. Furthermore, pathological activation of TGFβ1-Smad3 signaling and apoptosis, essential pathophysiological causes of AA-induced nephropathy (AAN), were ameliorated by BZM, suggesting this mechanism may be involved in improving fibrosis in AAN. In conclusion, BZM directly inhibits renal fibrosis in CKD via suppression of TGFβ1-Smad3 signaling and is promising in terms of drug repositioning.

## Introduction

The prevalence of chronic kidney disease (CKD) continues to increase and urgent countermeasures are necessary for medical economic reasons. Despite the identification of multiple promising compounds from aggressive experimental verifications, there are few treatments available in clinics to prevent CKD progression. CKD is characterized by the deposition of a pathological fibrillar matrix in the potential space between tubules and peritubular capillaries, which contains fibrillar collagen I and III^1^. Kidney fibrosis and fibrogenesis exacerbate CKD progression^2–4^ and are essential therapeutic targets.

Bortezomib (BZM) is a proteasome inhibitor used for the treatment of multiple myeloma (MM) and several recent studies have demonstrated that BZM attenuates renal impairment in patients with MM^5–7^. The International Myeloma Working Group published BZM-based regimens for the management of myeloma-related renal impairment in 2016^8^. Generally, renal improvement in MM by BZM is considered to be due to MM remission and amelioration of cast nephropathy, which is a direct consequence of the high serum concentration of immunoglobulin free light chains (FLCs).

Recently, several studies in experimental animal models reported that BZM prevents tissue fibrosis in lung, liver and skin via suppression of TGF-β1^9–12^. TGF-β1 is a profibrotic cytokine found in chronic renal diseases and is as a central mediator of tubulointerstitial fibrosis^13–15^. However, the effect of BZM on renal fibrosis remains to be determined.

We recently reported a case of MM with severe renal injury requiring regular hemodialysis. The patient received monthly maintenance treatment with BZM and dexamethasone therapy for MM for two years after achievement of complete response. The patient was finally withdrawn from maintenance hemodialysis therapy^16^, suggesting that BZM may prevent fibrosis in the kidney.

This study investigated the effect of BZM in mice with aristolochic acid (AA)-induced nephritis (AAN), a model conventionally to study renal fibrosis and mediated by TGFβ1-Smad3 signaling^17^, to determine the effect of BZM on renal fibrosis beyond the frame of MM treatment.

## Results

### Bortezomib attenuated aristolochic acid I (AA)-induced renal dysfunction and albuminuria

**Figure 1:**
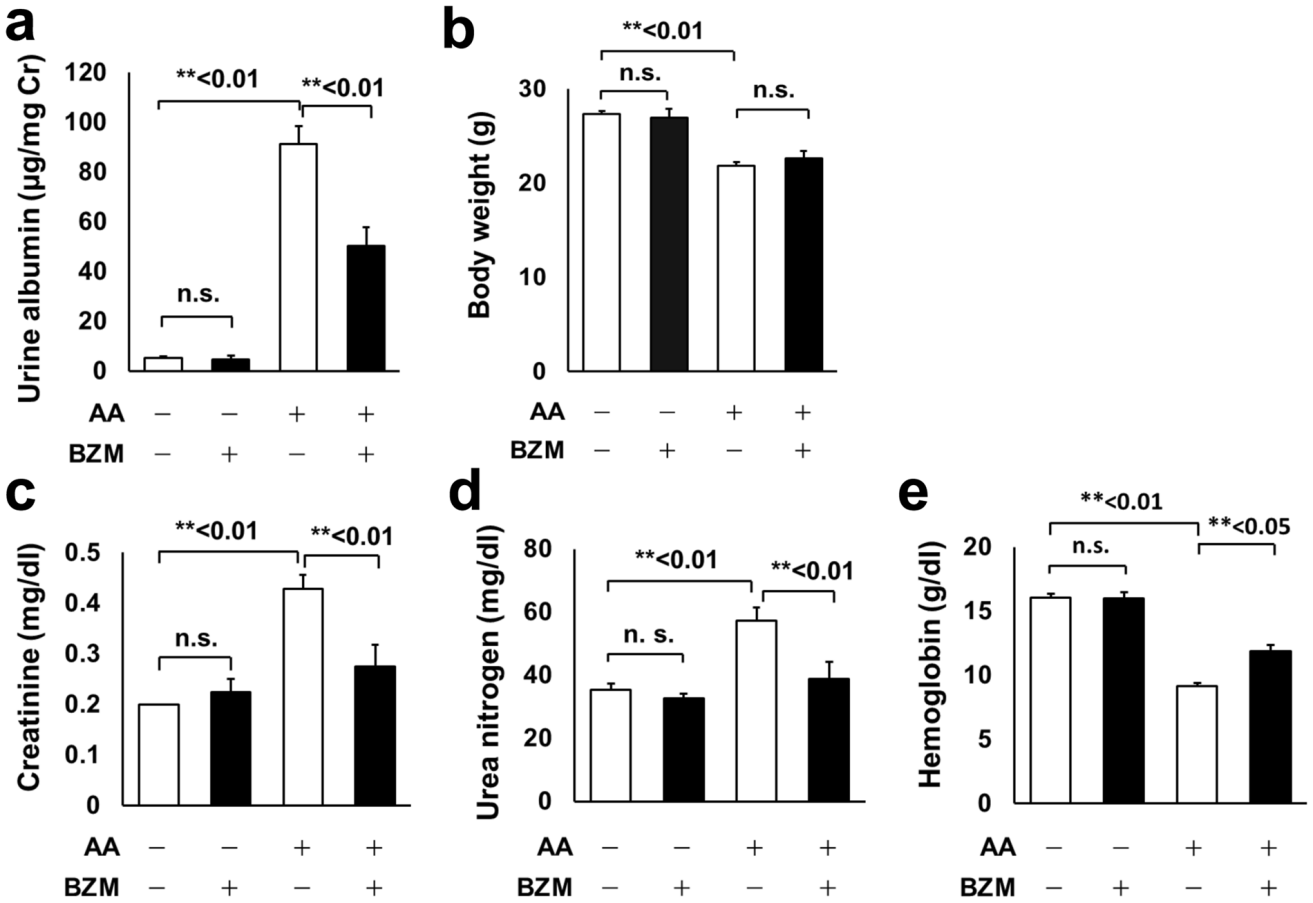
General characteristics and serological improvements in AAN mice with bortezomib treatment. (**a**) Albuminuria and (**b**) body weights of the AAN mice treated with or without BZM. BZM significantly improved albuminuria (**a**) but did not affect weight loss (**b**). (**c**) Serum creatinine, (**d**) Serum urea nitrogen, and (**e**) Hemoglobin of AAN mice treated with BZM. BZM significantly improved renal dysfunction and anemia induced by aristolochic acid. AA; aristolochic acid-1, BZM; bortezomib. Values presented are means ± SEM. **P < 0.01 (n = 5, 4, 7, 5).

We adopted the AAN model to investigate the effect of BZM on renal fibrosis. AAN was successfully reproduced by intermittently administering 3 mg/kg AA to C57BL/6J mice^17^. The intraperitoneal administration of BZM for 10 weeks significantly improved albuminuria induced by AAN (Fig. 1a). Body weight was reduced by AA administration, presumably due to drug toxicity or renal dysfunction and consistent with previous reports^17–20^. BZM did not affect weight loss (Fig. 1b). Serological data indicate that BZM significantly attenuated renal dysfunction (Fig. 1c–e and Table 1). Increased levels of serum creatinine (Cre) and urea nitrogen (UN) observed in AAN model mice were significantly attenuated following treatment with BZM (Fig. 1c,d). Metabolic acidosis was not apparent in AAN (Table 1). Interestingly, mice treated with BZM showed significant improvements in anemia (Fig. 1e).

**Table 1:** Blood biochemistry of aristolochic acid nephropathy mice treated with bortezomib.

AA	−	−	+	+
BZM	−	+	−	+
Hb (g/dl)	16.1 ± 0.3	16.0 ± 0.5	9.2 ± 0.2^†^	11.9 ± 0.5*
Hct (%)	47 ± 1	47 ± 2	27 ± 1^†^	35 ± 1*
UN (mg/dl)	35 ± 2	33 ± 1	57 ± 4^†^	39 ± 5**
Cre (mg/dl)	0.2 ± 0.0	0.2 ± 0.0	0.4 ± 0.0^†^	0.3 ± 0.0**
HCO_3_^−^ (mmol/l)	22 ± 1	24 ± 1	21 ± 1	22 ± 1

AA; aristolochic acid-1, BZM; bortezomib, Hb; hemoglobin, Hct; hematocrit, UN; urea nitrogen, Cre; creatinine. ^†^<0.01, ^‡^<0.05 compared with AA-BZM-group. **<0.01 compared with AA + BZM- group. (n = 5, 4, 7, 5).

### Bortezomib treatment improved AA-induced renal fibrosis

**Figure 2:**
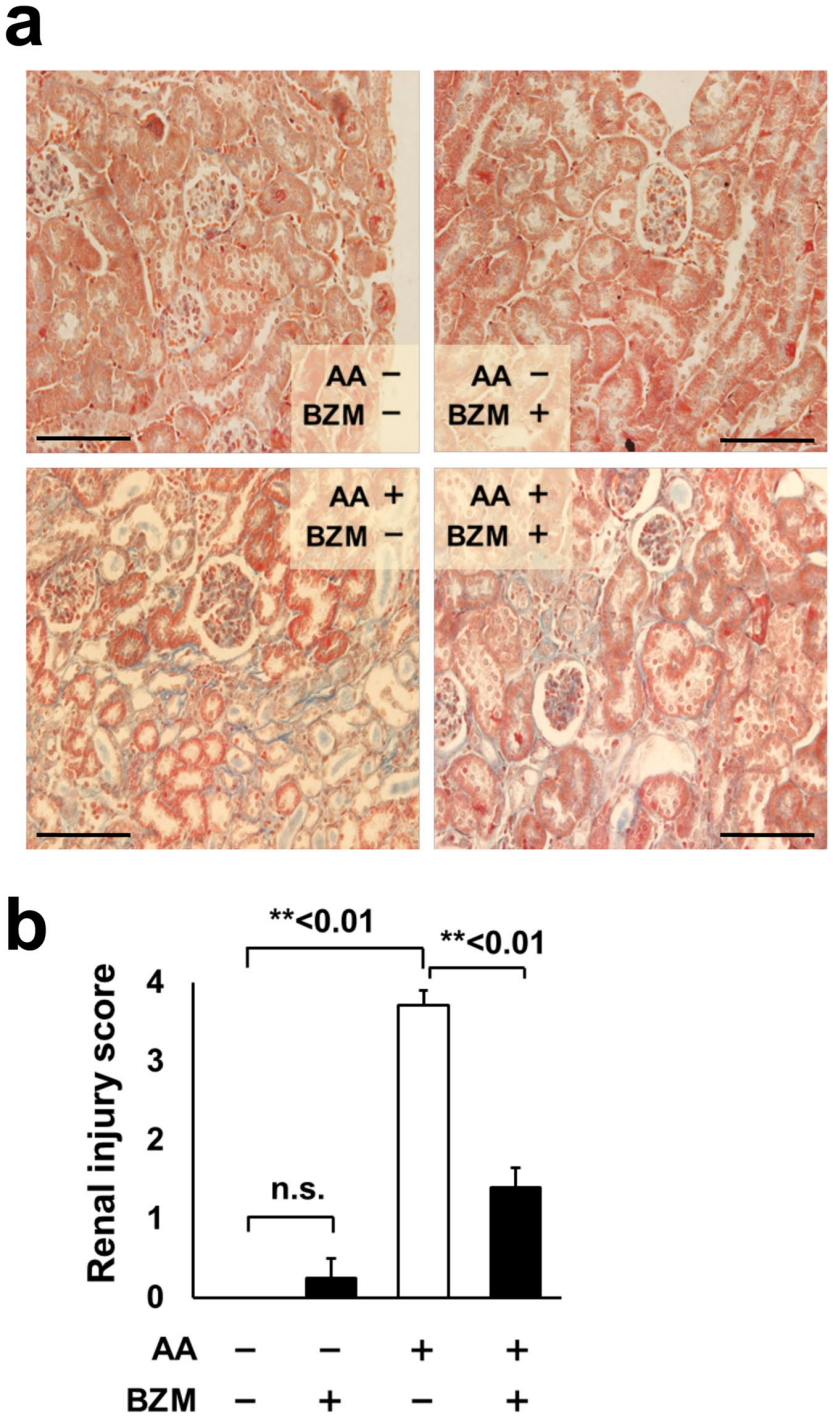
Histopathology of AAN mice treated with bortezomib. (**a**) Masson’s trichrome staining of kidneys from AAN mice with or without BZM treatment. BZM significantly improved fibrosis induced by AA. Magnification is 360x. (**b**) Jablonski’s renal injury score. BZM ameliorated renal injury score deteriorated by AA. AA; aristolochic acid-1, BZM; bortezomib. Values presented are means ± SEM. *P < 0.05; **P < 0.01 (n = 5, 4, 7, 5).

We hypothesized that the renal improvement was mainly due to the amelioration of renal fibrosis because the main feature of AAN is progressive renal interstitial fibrosis. We investigated histopathological changes in the kidney and the expression of renal fibrosis markers in AAN mice. Masson’s trichrome staining showed an apparent increase of interstitial fibrotic regions in AAN kidneys. BZM dramatically reduced the fibrotic regions induced by AA (Fig. 2a). Jablonski’s renal injury scores supported the histological finding that BZM significantly ameliorates interstitial fibrosis in AAN kidney (Fig. 2b).

### Tubular injury markers, fibrosis markers, and activation of TGF-β1-Smad3 signaling are ameliorated by BZM treatment

**Figure 3:**
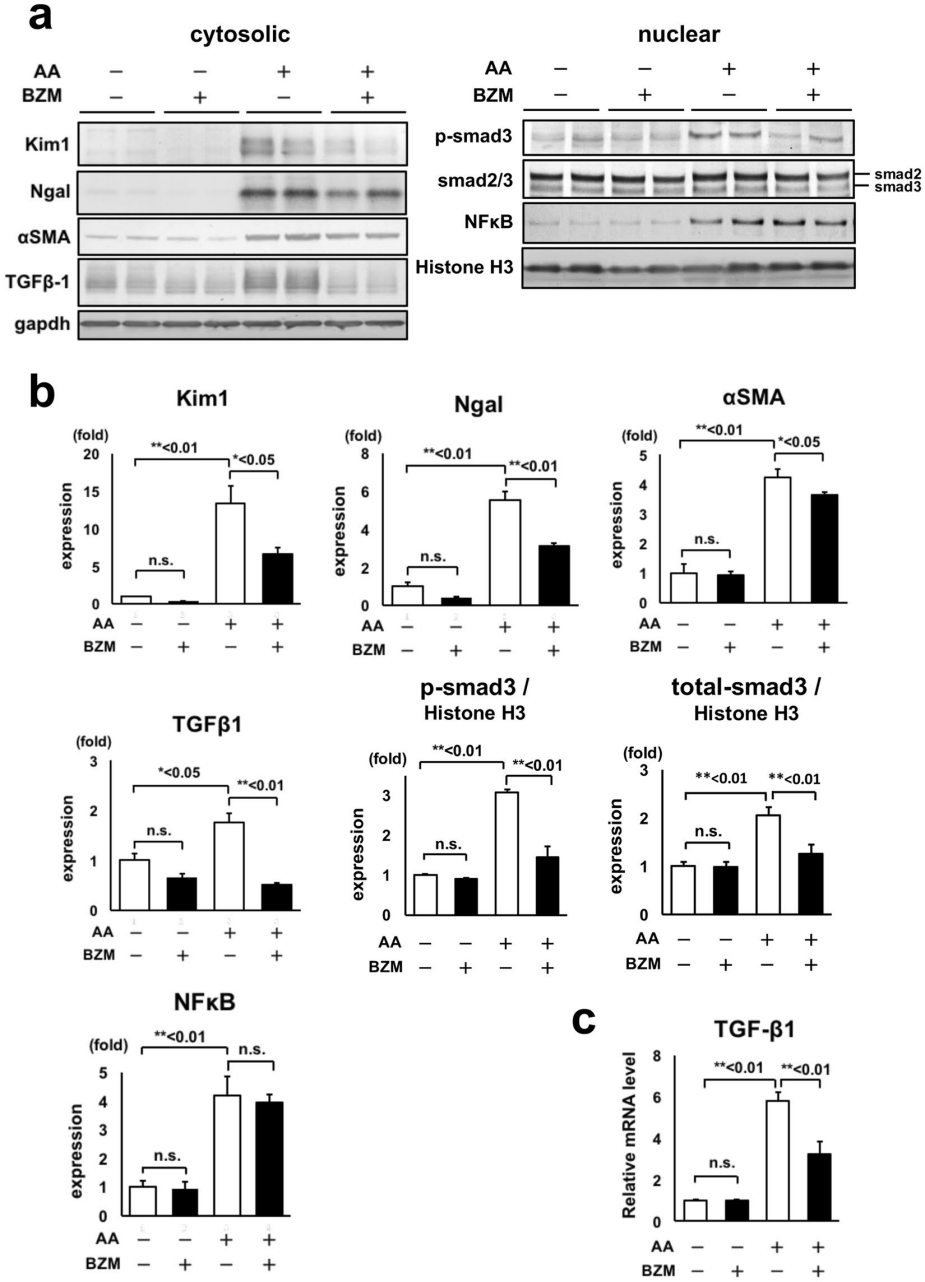
Bortezomib increased the expression of tubular injury markers, renal fibrosis marker, and TGF-β1 signaling induced by AA administration. (**a**) Representative immunoblot of Kim1, Ngal, αSMA, TGF-β1, phosphorylated Smad3, Smad2/3, and NFκB in AAN mice kidneys treated with BZM. (**b**) Densitometry analysis of the proteins analyzed in (**a**). (**c**) mRNA levels of TGF-β1 analyzed by quantitative RT-PCR. The mRNA level was significantly improved by BZM. Values presented are means ± SEM. *P < 0.05; **P < 0.01 (n = 5, 4, 7, 4).

Neutrophil gelatinase-associated lipocalin (Ngal) and kidney injury molecule-1 (Kim1) are markers for tubular injury^21,22^, and their expression increases in severe tubular injuries. α-Smooth muscle actin (α-SMA) reflects renal fibrosis levels. Therefore, we evaluated the expressions of Kim1, Ngal, and α-SMA in AAN (Fig. 3a,b). In the AAN groups, the expressions of all three genes were significantly increased after administering AA, and the expressions decreased with BZM treatment, thereby supporting the histological improvements observed.

The development of renal fibrosis in AAN is dependent on TGF-β1-Smad3 signaling^23^. We thus hypothesized that BZM protected renal fibrosis via suppression of TGF-β1 as well as the anti-fibrotic mechanisms of BZM in other organs. To confirm this, we investigated the protein expression and mRNA levels of TGF-β1 in AAN mouse kidneys. TGF-β1 protein levels elevated by the administration of AA were significantly improved by BZM (Fig. 3a,b). TGF-β1 mRNA was suppressed with BZM treatment as well (Fig. 3c). We also investigated the expression of Smad3, the downstream target of TGF-β1, to confirm the involvement of TGF-β1 in the BZM-mediated improvement of renal fibrosis. Smad3 is phosphorylated by TGF-β1 and translocated into the nucleus, which promotes gene transcription of various fibrosis-related factors^24^. The nuclear fraction of AAN kidneys treated with BZM showed a significant decrease in phosphorylated Smad3 compared with AAN alone, indicating that TGF-β1 signaling in AAN kidney is suppressed by BZM treatment.

### Cell cycle arrest and apoptosis induced by AA are ameliorated by BZM treatment

**Figure 4:**
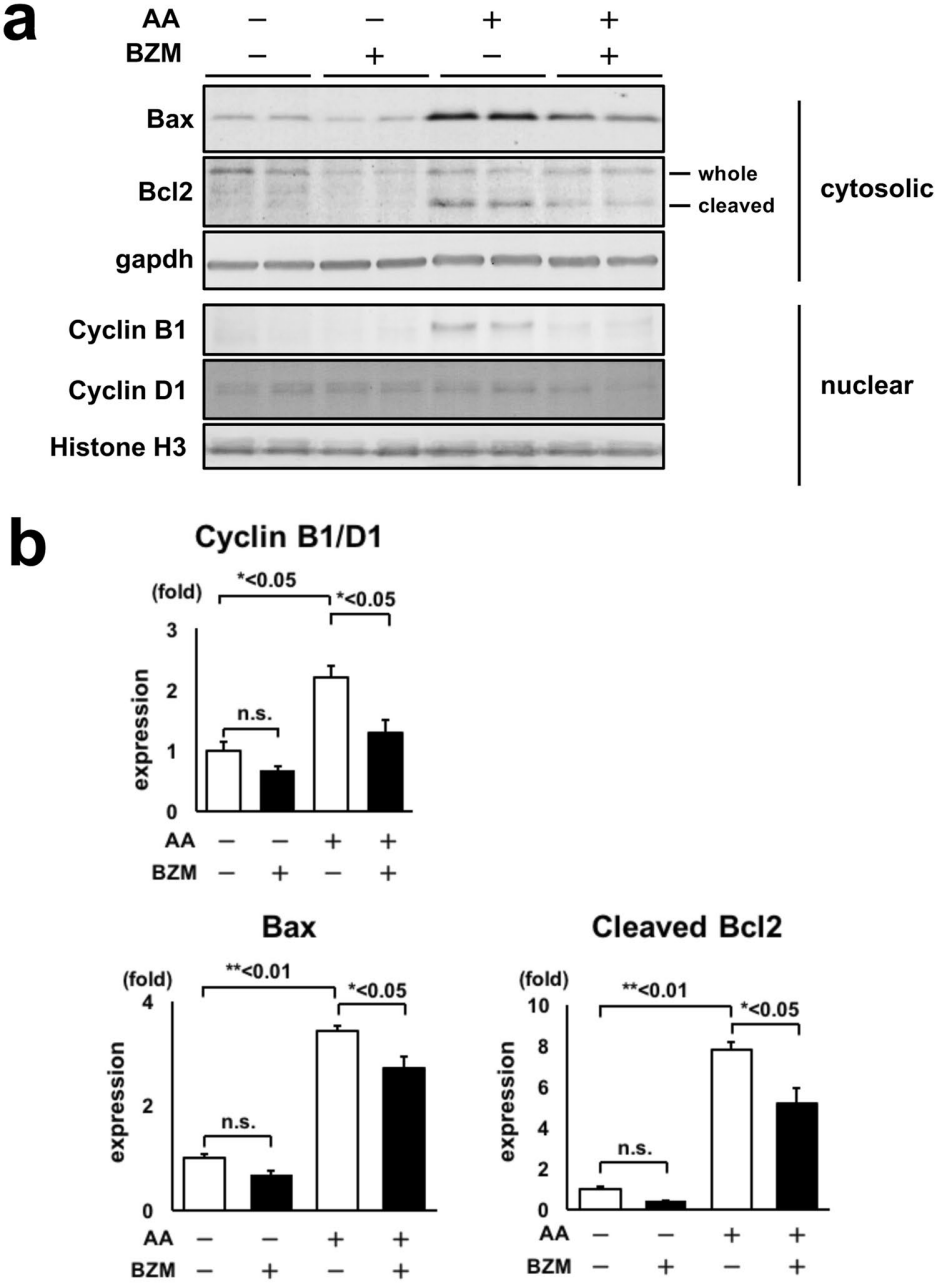
Cell cycle arrest and apoptosis induced by AA were ameliorated by BZM treatment. (**a**) Representative immunoblot of Bax, Bcl2, cyclin B1 and cyclin D1 in AAN mice kidneys treated with BZM. (**b**) Densitometry analysis of the proteins analyzed in (**a**). BZM significantly suppressed AA-induced increases in the expression of Bax, cleaved Bcl2, and ratio of cyclin B1 to cyclin D1. Values presented are means ± SEM. *P < 0.05; **P < 0.01 (n = 4).

We investigated whether BZM affects cell cycle and apoptosis because one of the major mechanisms of kidney fibrosis is epithelial cell cycle G2/M arrest^25^. Although BZM induces apoptosis and leads to cell death in tumor cells, the effect of BZM in non-tumor cells is controversial. Thus, we evaluated protein expression levels of cyclin B1 and D1 in crude nuclear fraction samples to estimate the cell cycle. In general, cyclin B1 corresponds to the G2/M phase, and cyclin D1 is expressed from G1 through the M phase. The ratio of cyclin B1 to cyclin D1 is generally used to estimate cell cycle^25^. The ratio of cyclin B1/D1 was increased in AAN, which is consistent with a previous report^26^ (Fig. 4a and b). This change was reversed by BZM treatment, suggesting that the majority of kidney cells in AAN are in G2/M arrest, and BZM prevents this change. To investigate whether BZM affects apoptosis, we evaluated protein expression levels of Bax and Bcl2 (Fig. 4). Bax forms a heterodimer with Bcl2 and activates apoptosis. Protein expression levels of Bax were significantly increased in AAN and were reduced after BZM treatment. Bcl2 cleavage, which represents cell apoptosis^27^, was increased in AAN and decreased after BZM treatment (Fig. 4a,b). Altogether, these data show that AA administration induces cell cycle arrest and apoptosis, which is ameliorated by BZM.

## Discussion

In this study, we demonstrated that BZM suppresses the activation of TGF-β1-Smad3 signaling and has an anti-apoptotic effect in AAN, thereby reducing proteinuria and ameliorating renal function and histopathological changes in the kidneys. Although the improvement of renal function in MM patients is generally considered to be an indirect effect of MM remission and the reduction of FLCs, our results clearly show that BZM directly prevents renal fibrosis in an AAN mouse model. This suggests that BZM may be applicable to CKD with etiologies other than MM. Treatments for renal fibrosis have been extensively investigated. However, investigations on the role of proteasome inhibition in preventing fibrosis have just begun. Upregulation of TGF-β1 reportedly causes the progression of renal fibrosis^13–15^. In a recent study, the proteasome inhibitor MG132 inhibited myofibroblastic transformation induced by TGF-β1 *in vitro*, which indicates that proteasome inhibitor has anti-fibroblastic effects^28^, supporting our findings.

Although proteasome inhibition is a promising strategy for sensitizing cancer cells to apoptosis, the anti-apoptotic effects of BZM on damaged non-tumor cells has been controversial. Rapino *et al*. reported that BZM blocks taxol-induced apoptosis by inhibiting the G2/M transition and mitigating MCL-1 degradation^29^, thus indicating that the effect of BZM on the cell cycle can vary depending on the cytotoxic stimulus or cell type. The study results support our finding that BZM has an anti-apoptotic effect on AAN.

We also evaluated other molecules that interact with TGF-β1. AMP-activated protein kinase (AMPK) signaling and nuclear factor-erythroid-2-like 2 (Nrf2) improve renal fibrosis by suppressing TGF-β1 or inflammation-related signaling^30,31^. However, AMPK and Nrf2 were not affected in our model (Supplementary Figure 1). Although Bcl-2 phosphorylation and cleavage mediate the anti-apoptotic function of BZM, we could not detect a significant phospho-Bcl-2 signal in our model, presumably owing to the insufficient sensitivity of the antibodies.

TGFβ/Smad3 signaling reportedly induces G2/M arrest and apoptosis^32,33^, indicating the anti-apoptotic effect that might be a direct consequence of TGFβ/Smad3 suppression. However, evaluating the interaction between these two pathways will require further study.

In this study, we mainly investigated the effect of BZM on AA-induced renal fibrosis. In other kidney injury models such as the unilateral ureteral obstruction (UUO) model, renal fibrosis is reportedly mediated by the activation of TGF-β1 signaling^34^. The results of treating the UUO model with BZM are shown in Supplementary Figure 2. BZM treatment clearly suppressed protein expressions of TGF-β1 and phosphorylated Smad3 in the UUO and AAN models. In addition, there was a trend toward reduced protein expressions of αSMA, Kim1, and Ngal. Histological findings improved after BZM treatment in the UUO model (Supplementary Figure 2). Although the expressions of the kidney injury markers α-SMA, Kim1, and Ngal tend to be reduced by BZM treatment, their effects on the UUO model are milder than those on the AAN model. The stimuli-induced by tissue damage may be stronger in the UUO model, or the differences may be a matter of optimal dosage or timing of BZM administration. Given that MG132 suppressed the TGFβ signaling pathway but did not ameliorate tubulointerstitial fibrosis in the rat UUO model^35^, it may be difficult to evaluate the effect of BZM on tissue amelioration in the UUO model. It remains unclear whether BZM can improve established fibrosis in a more advanced stage of CKD.

Interestingly, BZM significantly improved AAN-induced anemia. Severe anemia is often induced by administering AA, and there are several reports that investigate the underlying mechanism^36,37^. A speculative association between erythropoietin (EPO)-producing cells and fibrosis has been suggested^38^, and Asada *et al*.^39^ recently reported that EPO-producing cells in healthy kidney and scar-producing myofibroblasts originate from the same extrarenal cells during fibrosis. Although fibrogenesis from EPO-producing fibroblasts could be inhibited by BZM, further validation remains necessary to prove the hypothesis.

In conclusion, our study showed that the simultaneous administration of BZM with AA has an anti-fibrotic effect in kidney and prevents CKD progression by suppressing the expression of TGF-β1/Smad3 signaling and decreasing apoptosis. BTZ is an effective treatment for MM^40^ and has some utility in treatment of post-transplant rejection^41,42^. Our findings reposition BTZ as a new drug for treatment of renal fibrosis and illustrate its potential as a promising new therapeutic option for CKD.

## Methods

### Statement

All experiments and methods were performed in accordance with relevant guidelines and regulations. All experimental protocols were approved by a named institutional/licencing committee. Specifically, all animal experiments were approved by the Institutional Animal Care and Use Committee of Tokyo Medical and Dental University.

### Mouse model and drug infusion study protocols

Experiments were performed on 8 weeks-old male C57BL/6J mice, purchased from Japan SLC, Inc. All foods were obtained from Oriental Yeast Co., Ltd.

Aristolochic acid-I (3 mg/kg body weight, Sigma-Aldrich, St. Louis, MO) and BZM (0.5 mg/kg body weight, Funakoshi, Tokyo, Japan) were injected into each mouse intraperitoneally twice a week for 10 weeks. Then, spot urine was collected and kidneys were extracted after sacrifice.

### Blood and urinary analysis

Blood was collected from the venous plexus near the mandible just before sacrifice. Blood was analyzed by iSTAT EC8+ (Abbott, Inc. Abbott Park, IL). Urine albumin levels were analyzed by Lbis® Albumin Mouse ELISA Kit (Shibayagi, Gunma, Japan). Urine creatinine levels were analyzed by a LabAssay^TM^ Creatinine kit (Wako, Osaka, Japan).

### Histological analysis

The kidneys were fixed in 10% Formalin Neutral Buffer Solution (Wako, Osaka, Japan) and embedded in paraffin. Sections were stained with Masson’s trichrome stain, and images of the section were captured at 360× magnification. Jablonski’s renal injury score was used for the histopathologic assessment^43^.

### RT-PCR

Total RNA from mouse kidney was extracted using TRIzol reagent (Invitrogen, Carlsbad, CA). The total RNA was reverse-transcribed using Omniscript reverse transcriptase (Qiagen, Hilden, Germany). Sequences for the RT-PCR primers for TGF-β1 employed are described in Table 2. The primers for GAPDH were purchased from TAKARA BIO (Takara, Shiga, Japan). Real-time PCR was performed in a single-step procedure using SYBR Premix Ex Taq™ II (Takara, Shiga, Japan) on a Thermal Cycler Dice Real Time System, model TP700/760 (Takara, Shiga, Japan).

**Table 2:** Primer sequence.

	Forward 5′ → 3′	Reverse 5′ → 3′
TGF-β1	TGACGTCACTGGAGTTGTACGG	GGTTCATGTCATGGATGGATGGTGC

### Immunoblotting

For the protein lysate of mouse kidney, kidneys were isolated and immediately frozen using liquid nitrogen. Kidneys were cut in half and lysed with NE-PER Nuclear and Cytoplasmic Extraction Reagents (Thermo Fisher Scientific, Yokohama, Japan) to obtain samples from nuclear and cytoplasmic fractions, respectively.

The primary antibodies used in this study were rabbit anti-αSMA antibody (Abcam, Inc. Cambridge, UK), goat anti-KIM-1 antibody (R&D Systems, Inc. Minneapolis, MN), goat anti-NGAL antibody (R&D Systems, Inc. Minneapolis, MN), mouse anti-TGF-beta 1 antibody (R&D Systems, Inc. Minneapolis, MN), rabbit anti-Bax antibody (Abcam, Inc. Cambridge, UK), rabbit anti-Bcl2 (D17C4) antibody (Cell Signaling, Danvers, MA), rabbit anti-phosphorylated Smad3 antibody (Cell Signaling, Danvers, MA), rabbit anti-Smad2/3 antibody (Cell Signaling, Danvers, MA)^39,43,44^, rabbit anti-NF-kappa B p65 (D14E12) XP antibody (Cell Signaling, Danvers, MA), rabbit anti-cyclin B1 antibody 4138 (Cell Signaling, Danvers, MA), rabbit anti-cyclin D1 antibody 2978 (Cell Signaling, Danvers, MA), rabbit anti-GAPDH antibody (Cell Signaling, Danvers, MA), and rabbit anti-Histone H3 antibody (Abcam, Inc. Cambridge, UK). Alkaline-phosphatase-conjugated anti-IgG antibodies (Promega Corporation, Fitchburg, WI) were used as secondaries for immunoblotting. WesternBlue (Promega Corporation, Fitchburg, WI) was used for the development of immunoblots. The relative intensities of immunoblot bands were determined by densitometry using ImageJ software (National Institutes of Health, Bethesda, MD).

### Statistical analysis

Statistical significance was evaluated using an un-paired t-test. All data were expressed as means ± SEM. One-way ANOVA followed by Bonferroni test was used for comparing more than three groups. *P* values of < 0.05 were considered statistically significant.

## Electronic supplementary material

Supplementary information
