# Supplementary material for: The proteasome inhibitor bortezomib attenuates renal fibrosis in mice via the suppression of TGF-β1
Source: Sci Rep. 2017 Oct 12;7:13086. doi: 10.1038/s41598-017-13486-x (PMC5638992; doi:10.1038/s41598-017-13486-x)
Supplement: Supplementary file 1 — Supplementary information [file 41598_2017_13486_MOESM1_ESM.pdf]

Online Supplement

The proteasome inhibitor bortezomib attenuates renal fibrosis in mice via the suppression of TGF-β1

Moko Zeniya[1], Takayasu Mori*[1], Naofumi Yui[1], Naohiro Nomura[1], Shintaro Mandai[1], Kiyoshi Isobe[1], Motoko Chiga[1], Eisei Sohara[1], Tatemitsu Rai[1], and Shinichi Uchida[1]

[1]Department of Nephrology, Graduate School of Medical and Dental Sciences, Tokyo Medical and Dental University, 1-5-45 Yushima, Bunkyo, Tokyo 113-8519, Japan

*Correspondence to: Takayasu Mori

Department of Nephrology, Graduate School of Medical and Dental Sciences, Tokyo Medical and Dental University

1-5-45 Yushima, Bunkyo, Tokyo 113-8519, Japan

Tel: +81-3-5803-5214; Fax: +81-3-5803-5215; E-mail: tmori.kid@tmd.ac.jp

Materials and Methods

Statement

All experiments and methods were performed in accordance with relevant guidelines and regulations. All experimental protocols were approved by a named institutional/licencing committee. Specifically, all animal experiments were approved by the Institutional Animal Care and Use Committee of Tokyo Medical and Dental University.

Mouse model and drug infusion study protocols

Experiments were performed on male 8 weeks-old C57BL/6 mice, purchased from Japan SLC, Inc. All foods were obtained from Oriental Yeast Co., Ltd.

Unilateral ureteral obstruction (UUO) or sham surgery was performed under somnopentyl (Kyoritsu, Tokyo, Japan). The left ureter was visualized via a flank incision and ligated with a 5-0 Nylon thread (Matudaika Kogyo, Tokyo, JAPAN). The sham surgery was performed to control mouse group. Bortezomib (0.5 mg/kg body weight, Funakoshi, Tokyo, Japan) were injected in each mouse intraperitoneally once a day on day -1, 1 to 14 day from the operated date. Then, the kidneys were extracted after sacrifice.

Immunoblotting

For the protein lysate of mouse kidney, kidneys were isolated and immediately frozen using liquid nitrogen. Kidneys were cut in half and lysed with NE-PER Nuclear and Cytoplasmic Extraction Reagents (Thermo Fisher Scientific, Yokohama, Japan) to obtain samples from

nuclear and cytoplasmic fractions, respectively.

The primary antibodies used in this study were same as written in the main text. Additionally, mouse anti-Nrf2 (Abcam, Inc. Cambridge, UK), rabbit anti-pAMPK (Cell Signaling, Danvers, MA), rabbit anti-tAMPK (Cell Signaling, Danvers, MA) were used. Alkaline-phosphatase-conjugated anti-IgG antibodies (Promega Corporation, Fitchburg, WI) were used as secondaries for immunoblotting. WesternBlue (Promega Corporation, Fitchburg, WI) was used for the development of immunoblots. The relative intensities of immunoblot bands were determined by densitometry using ImageJ software (National Institutes of Health, Bethesda, MD).

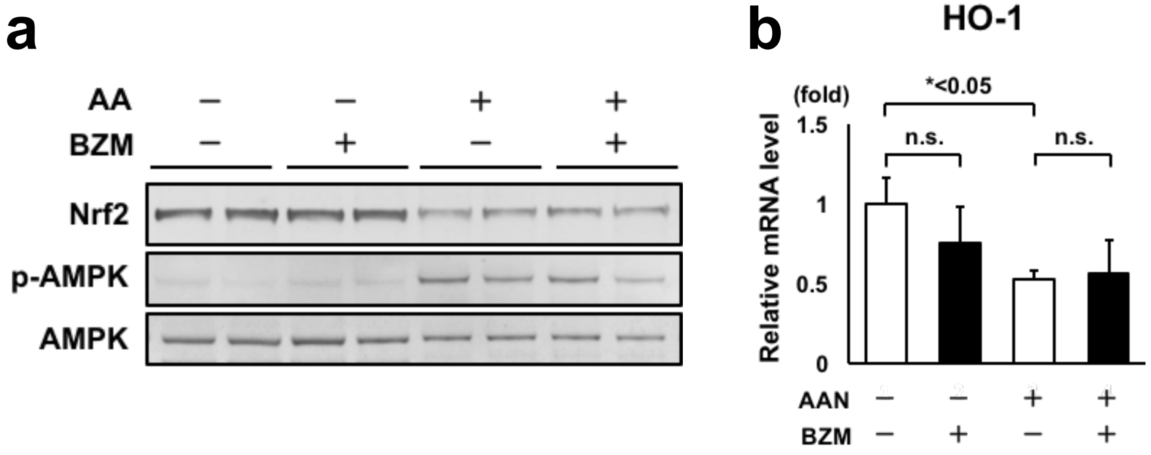

Supplementary figure 1. Neither Nrf2 signaling nor AMPK is affected by bortezomib treatment.

(a) Representative immunoblot of Nrf2, phosphorylated and total AMPK. Nrf2 is suppressed by AA administration but not affected by BZM treatment. Phosphorylation of AMPK is increased by AA but not affected by BZM. (b) HO-1 mRNA level in AAN mice kidneys analyzed by quantitative RT-PCR. HO-1 mRNA is significantly suppressed by AA but not affected by BTZ. Primer sequences of HO-1 are 5’-ATTGAGCTGTTTGAGGAGCTGC (Forward) and 5’-CACTGCCACTGTTGCCAACAGG (Reverse). Values presented are means ± SEM. **P* < 0.05. Nrf2: nuclear factor erythroid 2–related factor 2, AMPK: AMP-activated protein kinase, BZM: bortezomib.

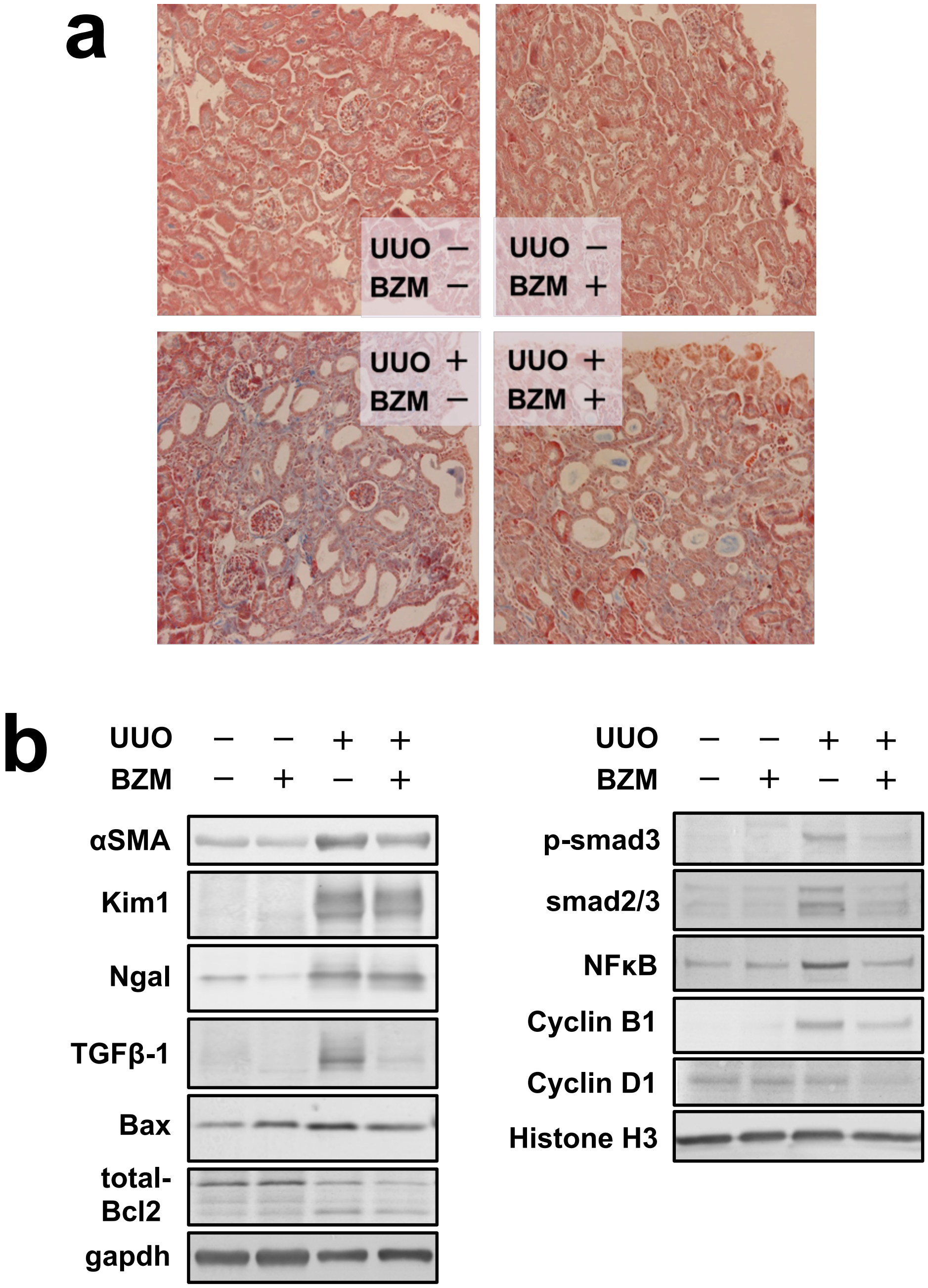

Supplementary figure 2. Histopathology and protein expressions related to kidney injury in UUO mice treated with bortezomib.

(a) Masson's trichrome staining of kidneys from UUO mice with or without BZM treatment. BZM improved fibrosis induced by UUO. Magnification is 360x. (b) Representative immunoblot of proteins related to kidney injury, apoptosis, and cell cycle in kidneys of UUO mice treated with BZM. Protein expressions of TGF-β1, pSmad3 are suppressed with BZM treatment in UUO, and those of αSMA, Kim1, Ngal tend to be reduced.

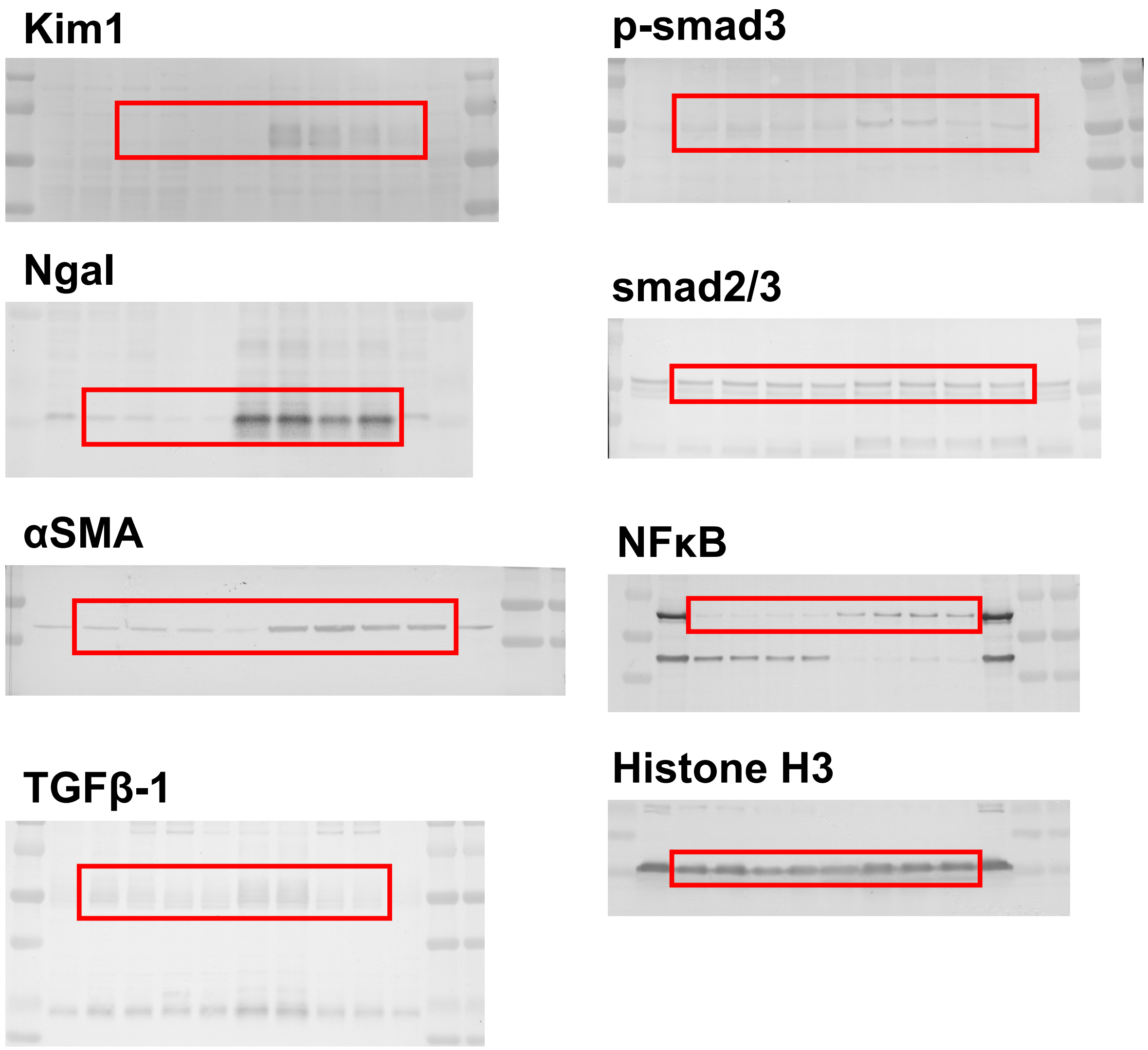

Supplementary Figure 3

Original western blots for the images shown in Figure 3. The cropped images are highlighted in the red lines.

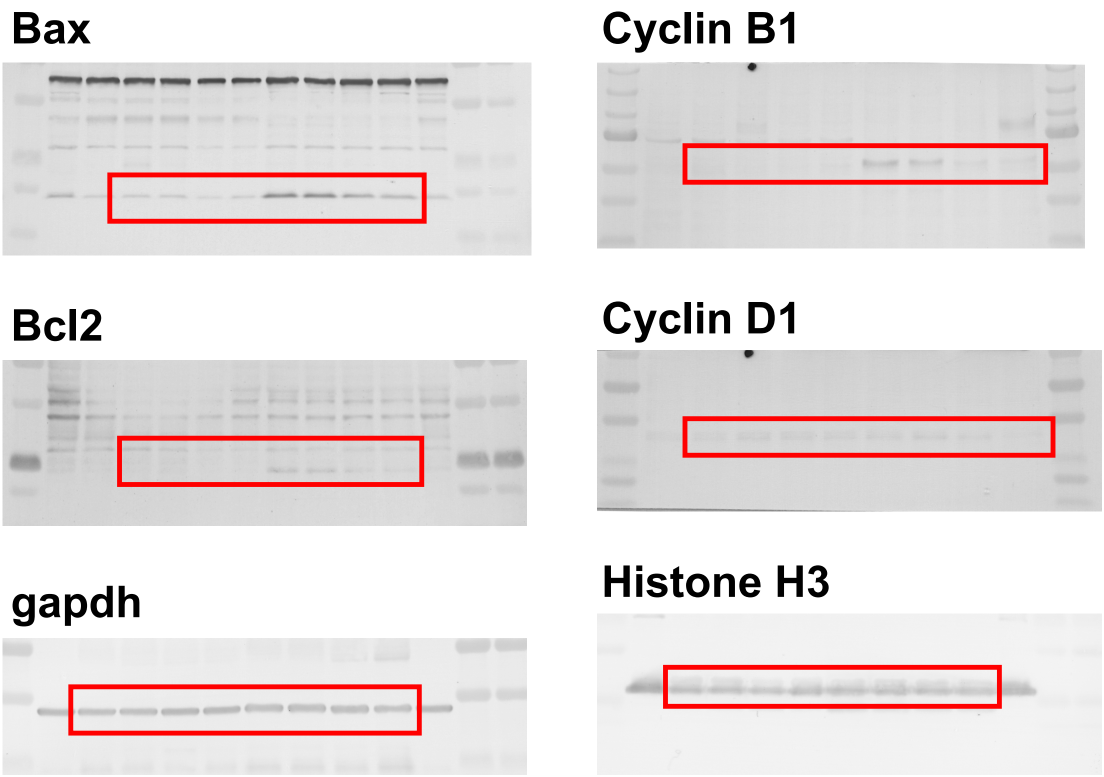

Supplementary Figure 4

Original western blots for the images shown in Figure 4. The cropped images are highlighted in the red lines.

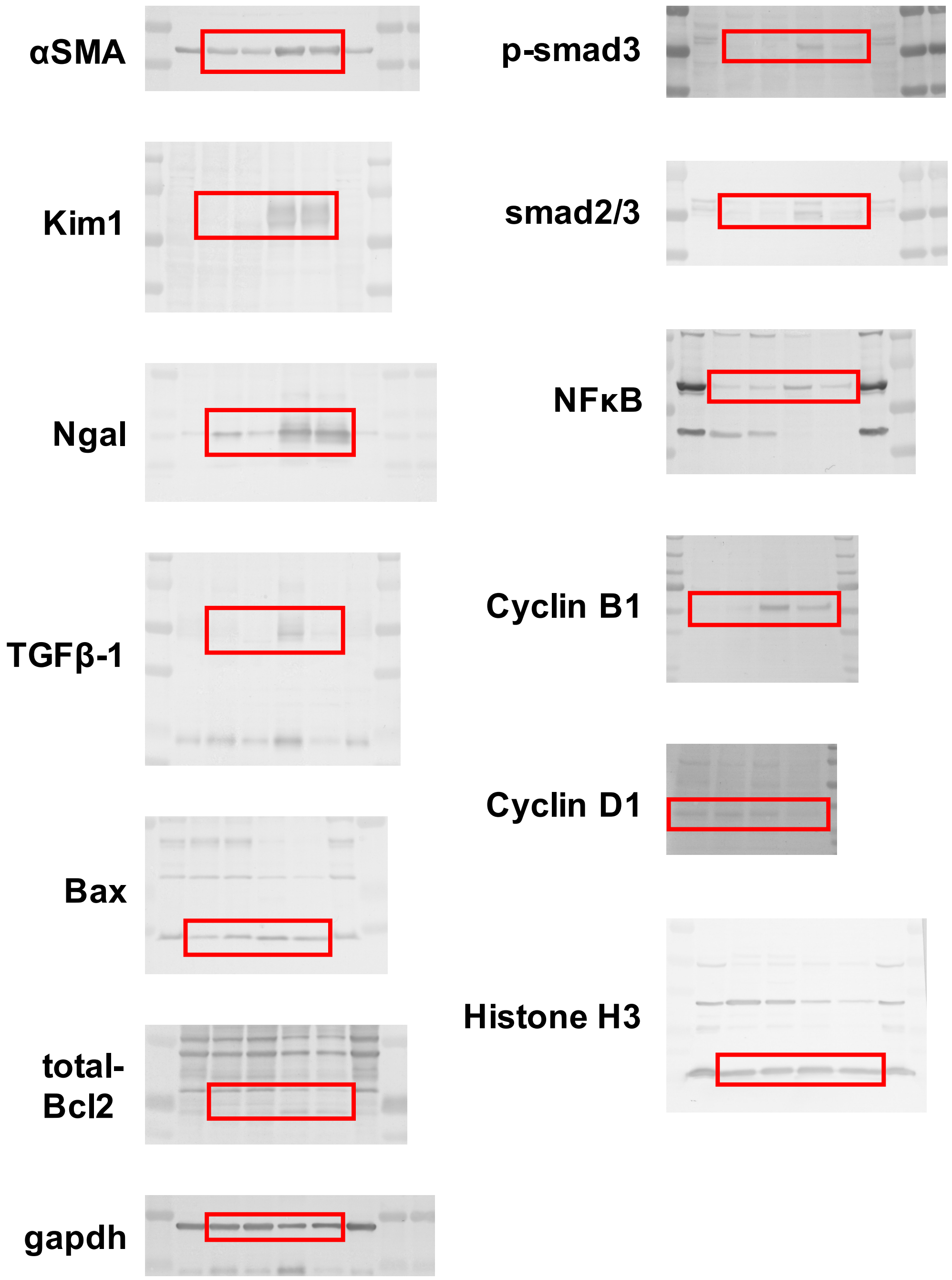

Supplementary Figure 5

Original western blots for the images shown in Supplementary figure 2. The cropped images are highlighted in the red lines.
